# Biomechanical Regulation of Hematopoietic Stem Cells in the Developing Embryo

**DOI:** 10.1007/s43152-020-00027-4

**Published:** 2021-01-26

**Authors:** Paulina D. Horton, Sandeep P. Dumbali, Krithikaa Rajkumar Bhanu, Miguel F. Diaz, Pamela L. Wenzel

**Affiliations:** 1 Department of Integrative Biology & Pharmacology, McGovern Medical School, University of Texas Health Science Center at Houston, 6431 Fannin St, MSB 4.130, Houston, TX 77030, USA; 2 Center for Stem Cell and Regenerative Medicine, The Brown Foundation Institute of Molecular Medicine, University of Texas Health Science Center at Houston, Houston, TX 77030, USA; 3 Immunology Program, MD Anderson Cancer Center UT Health Graduate School of Biomedical Sciences, Houston, TX 77030, USA

**Keywords:** Biomechanical force, Hematopoietic stem cells, Hematopoiesis, Mechanobiology, Mechanotransduction

## Abstract

**Purpose of Review:**

The contribution of biomechanical forces to hematopoietic stem cell (HSC) development in the embryo is a relatively nascent area of research. Herein, we address the biomechanics of the endothelial-to-hematopoietic transition (EHT), impact of force on organelles, and signaling triggered by extrinsic forces within the aorta-gonad-mesonephros (AGM), the primary site of HSC emergence.

**Recent Findings:**

Hemogenic endothelial cells undergo carefully orchestrated morphological adaptations during EHT. Moreover, expansion of the stem cell pool during embryogenesis requires HSC extravasation into the circulatory system and transit to the fetal liver, which is regulated by forces generated by blood flow. Findings from other cell types also suggest that forces external to the cell are sensed by the nucleus and mitochondria. Interactions between these organelles and the actin cytoskeleton dictate processes such as cell polarization, extrusion, division, survival, and differentiation.

**Summary:**

Despite challenges of measuring and modeling biophysical cues in the embryonic HSC niche, the past decade has revealed critical roles for mechanotransduction in governing HSC fate decisions. Lessons learned from the study of the embryonic hematopoietic niche promise to provide critical insights that could be leveraged for improvement in HSC generation and expansion ex vivo.

## Introduction

Hematopoiesis is the process of generation and maintenance of the various cells of the blood and immune systems [1]. Hematopoiesis originates with hematopoietic stem cells (HSCs), which differentiate into a diverse array of cell lineages and are able to self-renew, allowing them to continually replenish short-term progenitors and mature blood cells [2, 3]. This process is tightly regulated to prevent aberrant hematopoiesis, which can lead to hematologic disorders and malignancies [4]. Due to their inherent abilities to self-renew and differentiate, HSC transplantation has become the standard of care for many hematologic disorders [5]. However, the high risk of morbidity and mortality due to microbial infections, graft rejection, immunodeficiency, and organ failure has limited the efficacy and accessibility of this treatment [6, 7]. This, along with the unavailability of human leukocyte antigen (HLA) matched HSC donors for many patients, has led to an aggressive search for alternative sources of HSCs [8–11]. Yet, successful de novo generation of HSCs outside the body remains challenging [11, 12]. These efforts have primarily produced primitive HSC populations with skewed hemogenic potential, poor engraftment, and exhaustible contribution to the blood system [8–12]. Those that have produced human HSCs required maturation in vivo [8, 13]. The most successful in vitro models that have produced HSCs from endothelial and pluripotent sources have enforced expression of genes encoding transcription factors, in parallel with co-cultures producing vascular-niche angiocrine factors [14]. These reports support the importance of the niche in HSC specification and competence to self-renew. Accordingly, researchers have begun looking at the development of HSCs during embryogenesis to provide insight that could improve HSC generation and expansion ex vivo [11, 12].

In the developing embryo, hematopoiesis occurs dynamically over time at multiple sites [15, 16]. The first wave of hematopoiesis, known as primitive hematopoiesis, begins at embryonic day 7.5 (E7.5) in the blood islands of the yolk sac (YS) [17, 18]. In early embryos, blood circulation has a significant impact on vascular remodeling of the yolk sac. Interestingly, entry of blood cells into the blood stream increases hematocrit and blood viscosity, thereby elevating hemodynamic forces in the yolk sac vasculature and further propagating vessel remodeling [19]. Primitive hematopoiesis is an intermediate stage resulting in the generation of erythroid and myeloid progenitors but does not contribute to the adult HSC population [18, 20]. The primitive wave is followed by definitive hematopoiesis where the first HSCs emerge, which are capable of self-renewal and multi-lineage differentiation into hematopoietic cells of the adult [15]. While some sites of HSC development are still debated, numerous studies support that the first bona fide HSCs arise in the aorta gonad mesonephros (AGM) region of the embryo proper [15, 21]. Beginning at E10.5, HSCs bud off of the ventral wall of the dorsal aorta and move into the lumen of the vessel [22]. These HSCs arise from specialized endothelial cells in a process termed the endothelial-to-hematopoietic transition (EHT) that involves activation of transcriptional programs necessary for HSC development [23–25]. This is followed by substantial morphological change that allows newly specified HSCs to extravasate from the vascular wall into circulation to travel to the fetal liver [24–28]. The perivascular niche of the fetal liver serves as the next reservoir for HSC development [29]. At E11.5, HSCs begin to colonize the liver where they rapidly expand and differentiate into erythroid, myeloid, and lymphoid progenitors [16, 29–31]. Finally, HSCs migrate to the bone marrow (BM) at E17.5 where they remain throughout the lifetime of the individual [30, 32]. While the biochemical stimuli that regulate this process have been studied extensively, little is known about the role of biomechanical cues in these processes.

Blood circulation and HSC specification are intrinsically linked [33]. HSCs arise soon after initiation of the heartbeat at E8.25 [16]. The onset of circulation exposes the dorsal aorta of the AGM to hemodynamic forces that promote HSC development [33]. Many groups, including ours, have studied the mechanotransduction pathways triggered by biomechanical forces [18, 34]. Mechanobiology is a nascent concept in the history of hematology, and significant gaps in our knowledge regarding the mechanisms by which forces regulate HSC specification still remain (Table 1). Interestingly, recent studies have emerged demonstrating that forces exerted on a cell can have different effects on individual organelles such as the nucleus, mitochondria, and endoplasmic reticulum [35]. We and others are investigating the influence of mechanical force on developmental hematopoiesis and are seeking engineering solutions capable of harnessing these forces to improve HSC culture and disease modeling.

Progress in the past decade includes identification of some of the forces and associated cell signaling pathways that promote hematopoiesis; however, considerable work is required to understand how force can be leveraged for de novo HSC generation. In this review, we provide a brief overview of our current knowledge of mechanoregulation of developing HSCs as well as some of the primary challenges in this nascent field.

## Biomechanics of EHT

Throughout the lifetime of vertebrate animals, there is an intimate relationship between hematopoietic and endothelial cells [24]. In no place is this more evident than during embryogenesis in a process termed EHT [24, 36]. First described in 1998, this process involves activation of hematopoietic transcriptional machinery in specialized hemogenic endothelial (HE) cells [24, 37, 38]. The HE is a transient population of cells that can be found in the AGM, placenta, and umbilical vessels [39]. HE displays endothelial phenotypes and morphology and is capable of giving rise to both hematopoietic and endothelial cell types [36, 38, 40]. While HE cells morphologically resemble endothelial cells, they share many of the same cell surface markers expressed on hematopoietic stem and progenitor cells (HSPCs), including PECAM-1 (CD31), VE-cadherin (CD144), and CD34, making these two populations difficult to distinguish [36, 41]. Careful spatiotemporal characterization of hematopoietic clusters budding into the aorta during this transition has described a c-Kit^+^ CD31^+^ SSEA1^−^ population enriched for functional hematopoietic progenitors and stem cells [42].

Upon initiation of EHT, flat HE cells that are oriented along the vessel axis bend away from the vasculature until rounded [24, 40]. Imaging in zebrafish embryos reveals that endothelial cells on the aortic floor experience contraction and bending toward the subaortic space [26, 28]. The cells break tight junctions with neighboring endothelial cells and are then able to detach from the vascular wall as they downregulate endothelial cell surface markers and upregulate hematopoietic regulators [24, 40]. A critical driver of EHT is Runx1, a transcription factor required for HSC development [11, 24, 40, 43]. Runx1 is tightly regulated during development, and a number of studies have demonstrated that germline deletion of Runx1 not only results in an inability to form HSCs but also causes embryonic lethality [22, 40, 44]. While there is still little known about the specific complexes and networks in which this transcription factor functions, it is known to suppress endothelial cell transcription factors while upregulating hematopoietic factors such as Gata2, Myb, and Meis1 [24, 40]. Despite its identification 20 years ago, little is known about the molecular details of EHT [24]. Further complicating our understanding of EHT and HSC development is the observation that extravascular hematopoiesis can occur during embryogenesis [45]. Single-cell analysis, however, has made it possible to better investigate lineage hierarchies of HE cells during EHT [23, 41, 46–48]. Using this technology, researchers have demonstrated differences in the transcriptional landscape in the population of cells undergoing EHT, which is illustrated in part by changes in expression of the hematopoietic Wiskott-Aldrich Syndrome (*WAS*) gene [23]. These distinct transcriptome profiles account for populations of HSCs with differing hematopoietic activity and hematopoietic sublineage restrictions [23]. While studying the biochemical regulators of EHT will undoubtedly provide us with important insights into how this process works, it is important to note that changes in mechanical cues from the surrounding environment can also play a significant role in regulating EHT.

Morphological changes driven by contractility of the actomyosin cytoskeleton and detachment of tight junctions during EHT are critical aspects of HSC development [49, 50]. However, the mechanisms governing this cellular remodeling is poorly understood. In contrast, the mechanosensitivity of endothelial cells has been well documented, suggesting that clues to understanding the mechanics of EHT may lie in identification of the mechanosensitive components of HE cells. Adhesion molecules such as VE-cadherin, PECAM-1, VEGF-R2, and many other cell-cell adhesion-based receptors form adherens junctions responsible for transmitting force to adjacent cells [51–54]. These protein complexes participate in cell signaling and transcriptional control of endothelial cells [51, 52].

Both PECAM-1 and VEGF-R2/3 act as mechanotransducers in endothelial cells [51, 53]. VEGF-R2/3 is a mechanosensitive receptor necessary for hematopoietic development [55, 56]. As with PECAM-1 and VE-cadherin, VEGF-R2 can be found on HE cells and HSPCs and plays a crucial role in the development of the dorsal aorta and HSCs [36, 43]. During development, VEGF-R2 interacts with VE-cadherin to transduce mechanical signals into the cell [51, 53]. While its function in EHT has yet to be fully defined, the ability of VEGF-R2/3 to transduce mechanical stimuli into the cell and its necessity in HSC development makes it an attractive candidate as a possible regulator for EHT. PECAM-1 is a transmembrane cell adhesion molecule present on endothelial cells [53, 57]. It is concentrated at junctions between adjacent cells and contributes to the maintenance of the endothelial barrier [53, 57]. PECAM-1 signaling is crucial for mechanosensing in endothelial cells [51, 53]. Together with VE-cadherin, PECAM-1 helps promote flow-induced integrin activation, which is important for downstream mechanotransduction signaling and cytoskeletal remodeling [51, 53]. VE-cadherin is a vital component of adherens junctions and plays a key role in maintaining vascular integrity [51, 52, 58]. This transmembrane protein is first expressed around E7.5 in the mouse and is one of the first endothelial markers expressed [58]. However, VE-cadherin is also expressed in HSCs until E16.5, when it begins to decline [59]. Interestingly, VE-cadherin has been shown in multiple studies to be influenced by blood flow [51, 60, 61]. Phosphorylation of VE-cadherin is triggered by blood flow, resulting in increased vascular permeability [60]. The dynamic nature of adherens junctions may provide an explanation for some of the molecular changes that are observed during EHT. Together with PECAM-1 and VEGF-R2, VE-cadherin has been shown to activate MAPK, Akt, and PI3K signaling pathways, which have all been implicated in hematopoiesis [3, 62–64]. It is important to note that force has also been shown to activate mechanosensitive ion channels [3, 65]. In a zebrafish model analyzing the impact of blood flow on endothelial cell extrusion, the mechanosensitive cationic channel polycystic kidney disease 2 (*pkd2*) was found to be responsible for modulating endothelial cell extrusion independent of primary cilia [65]. This finding contrasts with other work that implicates primary cilia as mechanosensors in EHT [66]. While many unknowns regarding the biomechanics of EHT remain, improved tools in mechanobiology and heightened awareness of the importance of blood flow in this process ensures significant advancement in the coming years.

## Impact of Mechanical Force on Organelles

Eukaryotic cells, including HSCs, have dynamic and highly complex intracellular environments [35]. In this dynamic environment, organelles are trafficked and remodeled to carry out the various needs of the cell [35, 67, 68]. However, numerous membranous organelles and protein structures are confined to an extremely limited volume, exposing them to different physical stressors [35, 67]. In recent decades, our understanding of mechanobiology at the cellular level has greatly improved, but the study of mechanoregulation of sub-cellular components is in its infancy [35, 67]. While extremely little is known about the impact of mechanical stimuli on the organelles within HSCs, below we detail a subset of what is known from other cell types about the effects of physical force on two organelles that play central roles in HSC function, the nucleus and the mitochondria.

### Nucleus

The nucleus is the chief storage site for our genetic information and actively participates in sensing changes in mechanical load [35, 69–71]. Forces acting on a cell activate integrins, leading to a downstream signaling cascade through the actin cytoskeleton that transmits these forces to the nucleus [72]. This is possible because of the close relationship between the nucleus and the cytoskeleton, which is mediated by linker of the nucleoskeleton and cytoskeleton (LINC) complexes [70–75]. LINC complexes are believed to be the primary structures controlling nuclear mechanotransduction and are also important for regulating nuclear morphology and positioning, DNA repair, cell migration, and movement of chromosomes during meiosis [35, 70, 71, 75]. These complexes are composed of inner nuclear membrane SUN (Sad1/UNC-84) proteins and outer nuclear membrane nesprin proteins [35, 70, 71, 75]. SUN proteins interact with lamins and chromatin inside the nucleus while nesprins interact with various elements of the cytoskeleton in the cytoplasm [35, 70, 71, 75]. The role of nuclear mechanotransduction is not fully understood in regard to HSCs; however, in endothelial cells, nuclear mechanotransduction is critical for normal endothelial development [69, 76]. Force sensing by the nucleus has been shown to be necessary for endothelial cell polarization, which is important for endothelial cell rearrangements, shape changes, cell division, and cell extrusion, and delamination [65, 76]. In this study, endothelial cells were able to sense the direction of flow and induce polarization by mechanical displacement of their nucleus independent of primary cilia [76]. Primary cilia are thought to be crucial flow sensors in endothelial cells, as well as many other cell types, and this study suggests that they are unnecessary for early stages of endothelial morphogenesis [66, 76]. In the future, a priority should be to examine the impact of nuclear mechanotransduction on HSC development.

### Mitochondria

Mitochondria are dynamic organelles that have attracted great interest for their roles in HSC fate decisions and self-renewal capacity [77, 78]. They are able to form interconnected networks capable of remodeling in order to meet the ever-changing metabolic demands of the cell [78–80]. This is accomplished, in part, by mitochondrial fission and fusion which are essential for cell survival, mitochondrial redistribution, and maintenance of a healthy mitochondrial network [35, 78, 81, 82]. Dysfunctions in these processes can have profound effects on stem cell behavior, including loss of stemness and impairment of differentiation [67, 81, 83]. The main factors driving mitochondrial fission and fusion are Drp1, Mfn1/2, and Opa1 [81–83]. Mfn1/2 are large GTPases that are crucial for outer mitochondrial membrane fusion, while Opa1 is a protein in the dynamin family that is required for inner membrane fusion [81, 84]. Drp1 is a GTPase dynamin-related protein that assembles on the surface of mitochondria to mediate mitochondrial fission [81, 85]. Mitochondrial fusion is believed to be associated with increased oxidative phosphorylation and more efficient energy generation while mitochondrial fission is associated with glycolysis and allows for the elimination of damaged mitochondria [81, 86, 87]. Emerging evidence suggests an active role of mechanical force in regulating fission and fusion of mitochondria. Actin is able to bind and activate Drp1 and is recruited to fragmenting mitochondria [84]. Actin filaments have been shown to cycle on and off of a subset of mitochondria within a cell at any point in time in a actin-related protein (Arp) 2/3 and formin-dependent manner [84]. These filaments do not remain associated to the same subpopulation of mitochondria and instead cycle through different populations over time [84]. Actin assembles on the outer membrane of healthy, elongated mitochondria and promotes fission and inhibit fusion [84]. Subsequently, after mitochondrial fission actin disassembles from the fragmented mitochondria, which then rapidly refuse and reintegrate into the mitochondrial network [84]. Additionally, intracellular and extracellular forces applied to cells can induce mitochondrial fission at mechanically strained sites [80]. These findings are significant because they suggest that actin filaments continuously survey the surface of mitochondria, making mitochondria both mechanosensitive and subject to regulation by the various mechanical stimuli that act on HE cells and HSPCs [35]. Our research has shown that force associated with blood flow stimulates the cAMP/PKA/CREB signaling pathway in E10.5 HE cells [34]. This pathway activates a wide array of transcriptional cascades involved in HSC development, cellular metabolism, and mitochondrial biogenesis, further illustrating possible relationships between mitochondria and mechanosensing [34, 88]. Understanding links between force and metabolism is an important next step in unraveling the complexities of developmental hematopoiesis.

## Hemodynamic Forces in the Embryo

**Fig. 1:**
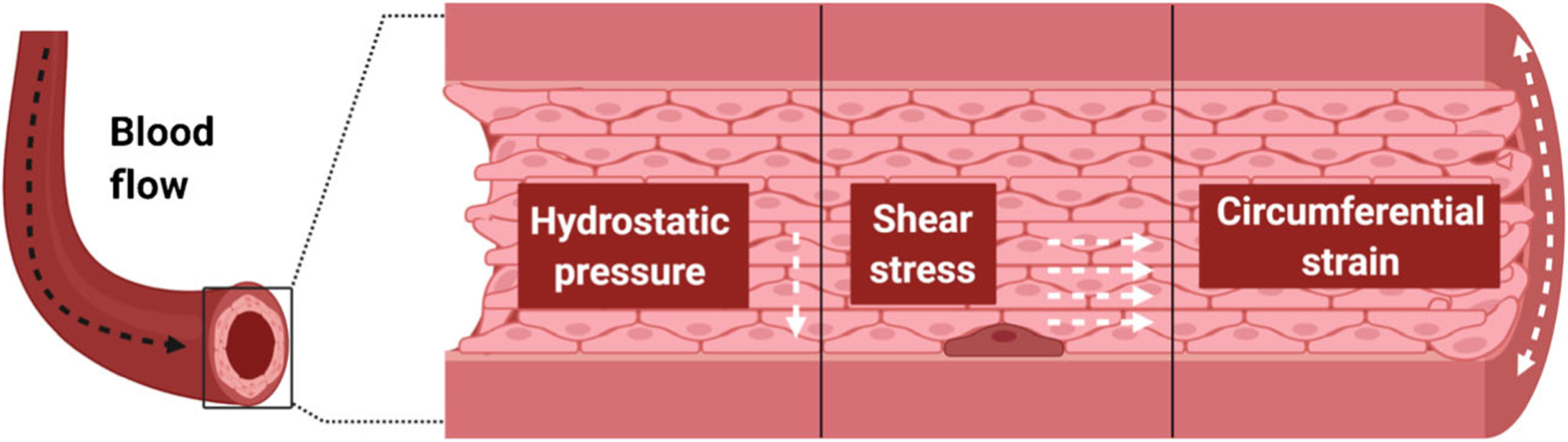
Hemodynamic forces within the embryonic aorta. Blood flow generates three forces that act on the vessel wall where hemogenic endothelial cells reside, including hydrostatic pressure, shear stress, and circumferential strain. Each force has distinct directionality which is depicted by white dashed arrows. HE cells reside within the vascular wall among the more numerous vascular endothelial cells

Similar to the internal forces acting within a cell, external forces in the microenvironment influence fate decisions of HSCs. Blood flow produces three types of forces including a frictional force referred to as wall shear stress (WSS), hydrostatic pressure, and circumferential strain or stretching (Fig. 1). Collectively, these forces are referred to as hemodynamic forces. These forces act on the endothelial cells lining the vascular wall and, to a lesser extent, the cells flowing through the bloodstream. In the perivascular space, hemodynamic force is even thought to transmit through interendothelial clefts or gaps in the basement membrane to expose the basolateral surface of cells outside the vessel to considerable shear stress equivalent to intraluminal forces [89]. It is also well established that paracrine signals produced by stimulated endothelial cells regulate cells associated with the vasculature, such as pericytes and muscle cells that envelop vessels. Indeed, these mechanical cues and the signals they produce are important for the maintenance of vascular integrity and homeostasis [90]. Importantly, these forces play an essential role in promoting HSC specification during embryogenesis. Below, we provide an overview of how hemodynamic forces contribute to development of the blood system and some of the signals that promote hematopoiesis.

### Wall Shear Stress

**Fig. 2:**
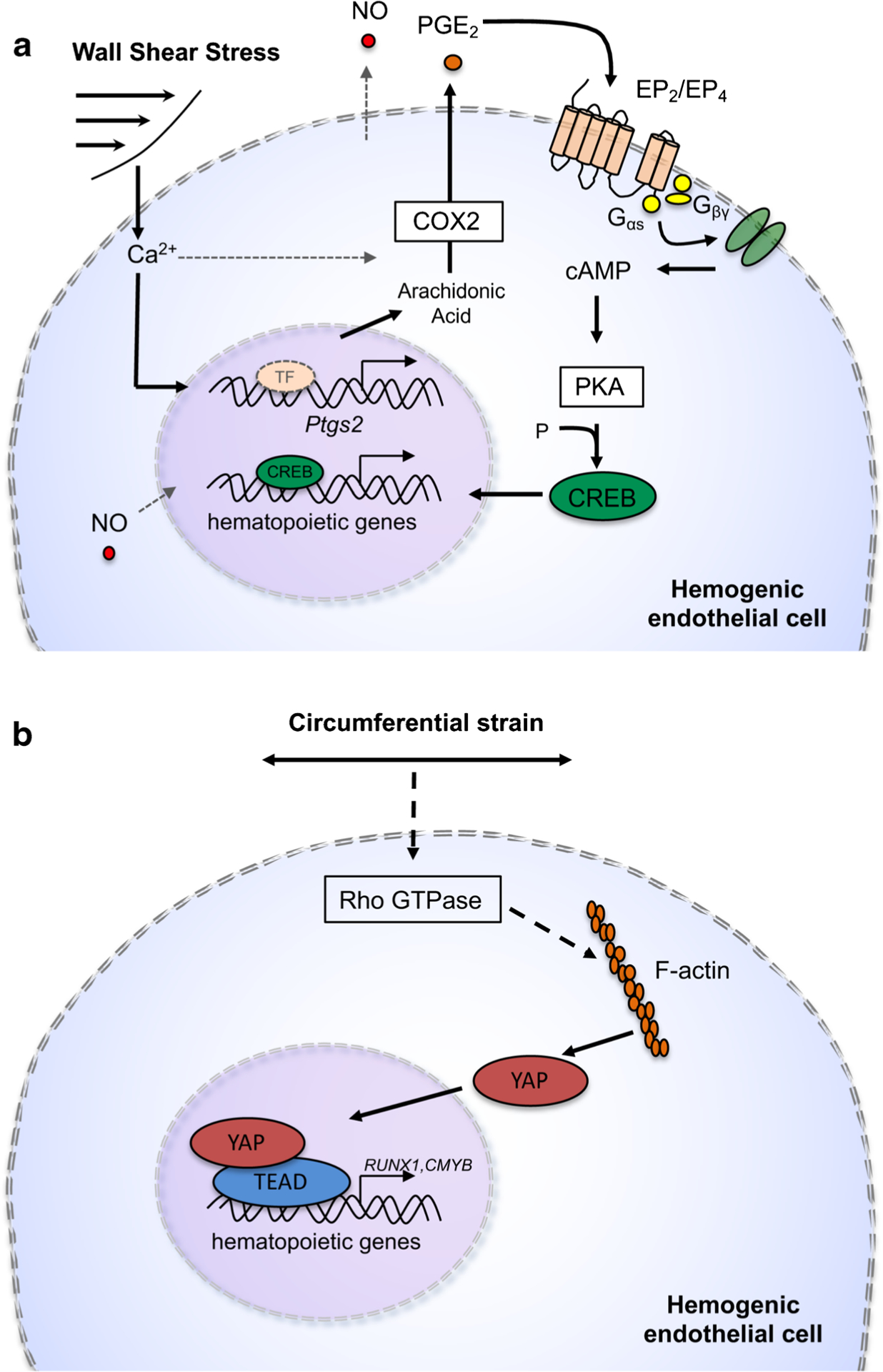
Models of intracellular signaling stimulated by biomechanical forces associated with blood flow. **a** Wall shear stress (WSS) triggers influx of cytosolic calcium ion (Ca^2+^) to amplify activity and transcript levels of the cyclooxygenase enzyme encoded by the *Ptgs2* gene. Elevated prostaglandin E_2_ (PGE_2_) production stimulates EP_2_/EP_4_ G protein coupled receptors to regulate the cyclic AMP (cAMP)-protein kinase A (PKA)-CREB signaling axis, which activates master regulators of hematopoiesis [34]. **b** Circumferential strain produces increased Rho GTPase activity that drives nuclear localization of YAP1 and transactivation of genes important for hematopoietic specification [103]

As stated above, WSS is defined as the frictional force generated parallel to the vessel wall by blood as it flows through the vasculature [33, 91]. Endothelial cells have evolved sophisticated mechanosensing machinery that allows them to detect distinct features of flow. Consequently, flow has profound effects on endothelial cell morphology and function [33, 91]. The flow magnitude, direction, amplitude, and frequency of pulsatile flow are all factors that can impact endothelial cell gene expression, angiogenesis, and lumen formation [65, 91]. The importance of blood flow in HSC development has also attracted attention in the past decade. In 2009, Adamo et al. first showed the requirement for biomechanical force in embryonic hematopoiesis [33]. Ex vivo culture of E10.5 mouse embryos under static or WSS conditions demonstrated that cells exposed to WSS express higher levels of Runx1 [18, 33]. The study also examined cardiac mutant embryos, which lack the sodium exchange channel Ncx1 required for the heartbeat and therefore lack blood circulation [92]. Consistent with results from ex vivo culture, Ncx1-null heartbeat mutants have significantly lower expression of Runx1 and exhibit a dramatic reduction in hematopoietic activity in the embryo proper [33]. Importantly, exposure of Ncx1-null embryos to WSS ex vivo rescues Runx1 expression and hematopoiesis [33]. Similarly, hematopoiesis could also be induced by mimicry of WSS with pharmacological induction of nitric oxide, a signaling pathway triggered by laminar flow [18, 33, 34]. This study established the foundation for subsequent reports detailing the mechanisms regulating these responses. For example, Diaz et al. demonstrated that WSS activates developmental pathways crucial for hematopoiesis, including Wnt and Notch, both of which are involved in the specification and generation of definitive HSCs from hemogenic endothelium [34, 43, 93] (Fig. 2a). WSS also stimulates calcium flux into the cytoplasm, leading to upregulated production of prostaglandin E_2_ (PGE_2_) [34]. PGE_2_ is a bioactive lipid of the prostanoid family that was previously shown to regulate HSC and progenitor cell self-renewal, survival, trafficking, and engraftment potential [94–99]. Increased PGE_2_, in turn, contributes to increased cAMP-PKA activity and CREB transcriptional activation of pathways that lie up-stream of Wnt and other pro-hematopoietic signals [34]. Taken together, these results link WSS to the regulation of biochemical and genetic pathways necessary for embryonic hematopoiesis [34]. As an extension of these studies, we have developed biomimetic platforms to address the effects of WSS and substrate elasticity on the steps after HSC emergence, wherein nascent HSCs traffic to the fetal liver for expansion [100]. While there are still numerous gaps in our knowledge of how WSS regulates HSC development, we know the most about WSS for its role in blood development.

### Circumferential Stress

Circumferential stress is a stretching force tangential and longitudinal to the vessel wall [101, 102]. As researchers work to unravel the mechanisms governing HSC specification in the embryo, circumferential strain has more recently been implicated as a potential regulator of this process. Lundin et al. has demonstrated Yes-activated protein (YAP) as a circumferential strain-induced regulator of HSPC formation [103] (Fig. 2b). YAP is a co-transcription factor that translocates to the nucleus in response to biomechanical force to contribute to cell fate decisions and has been shown to be regulated by Hippo and Wnt signaling, pathways important for HSC development [3, 43, 103, 104]. Human induced pluripotent stem cell (iPSC) derived hemogenic endothelial cells were exposed to WSS or circumferential strain conditions and, while both treatment conditions increased Runx1 expression, cells exposed to WSS exhibited a more significant increase in Runx1 than cells exposed to circumferential strain [103]. However, circumferential stress was a more potent activator of YAP signaling, and loss of YAP resulted in a significant decrease in Runx1 and cmyb expression [103]. Induction of YAP was mediated by elevated Rho-GTPase activity, suggesting that blood flow stimulates Rho-GTPase which then activates YAP-mediated HSPC production [103]. This is significant because Rho-GTPases are established mechanosensors, and this further supports a role for hemodynamic forces in embryonic hematopoiesis [3, 103, 105, 106]. Additionally, a recent conference abstract suggested that circumferential strain activates the mechanosensitive ion channel Piezo1 to stimulate EHT in hemogenic endothelium [107]. This response is believed to be brought about via induction of Dnmt3b, a DNA methyltransferase important for establishing DNA methylation patterns during development [107, 108]. While the involvement of Piezo in HSPC development still requires peer review, it will be interesting to see if connections emerge between mechanotransduction pathways and epigenetic programs.

### Hydrostatic Pressure

Lastly, hydrostatic or intraluminal pressure is the radial force exerted by blood against the walls of the vasculature [109]. Endothelial cells lining the vasculature are constantly exposed to hydrostatic pressure, and the study of biochemical and biomechanical responses to pressure in these cells is a fast-growing field [110]. Yet, our understanding of the relationship between pressure and developmental hematopoiesis remains poorly understood. Even the mechanisms governing pressure sensing have yet to be elucidated [111]. Recently, by employing use of a bioreactor capable of applying intermittent pressure, Kim et al. improved expansion of human HSPCs in vitro [112]. In these experiments, HSPCs were cultured for 7 days on a 3D fibrous scaffold and exposed to intermittent pressure to better recapitulate the in vivo microenvironment in the bone marrow [112]. HSPCs exposed intermittently to 20 kPa of pressure exhibited significant increases in total CD34^+^ hematopoietic cells and improvements in clonogenic potential based on in vitro colony forming and long-term culture-initiating cell assays [112]. In a similar experiment, Rodling and colleagues used a dynamic HSPC culture with perfusion in a macroporous hydrogel scaffold which mimicked the 3D bone marrow spongy architecture. This setup was effective in the maintenance and differentiation of CD34^+^ cells at early time points of HSPC culture [113]. While the mechanisms governing this response have not yet been defined, this work suggests that pressure may also play a role in HSC development and self-renewal. Currently, efforts focusing on the effects of hydrostatic pressure in the embryo are extremely limited, but future studies should be aimed at identification of factors involved in pressure sensing during embryonic hematopoiesis.

Overall, these findings advance our understanding of the mechanisms governing embryonic hematopoiesis and present intriguing areas of future study. By understanding the mechanisms that sense and propagate force, better tools can be engineered for hematologic disease modeling and de novo generation of HSCs.

## Challenges in the Field

Since their discovery in the 1950s, our understanding of HSCs has grown exponentially [114, 115]. The HSC niche and HSC function in the adult are well characterized and reviewed by thousands of articles, but little is known about the development of HSCs in the embryo. In recent years, the developmental programs governing HSC emergence in the embryo that improve in vitro culture and expansion have attracted greater attention [12, 116, 117]. With advances in our understanding of mechanobiology, study of the mechanosensors and mechanotransduction pathways activated in HSCs is more accessible [3, 18, 33]. Yet, much is still unknown regarding HSC mechanobiology and some persistent challenges have slowed progress.

### Measurement of HSC Frequency and Activity

Unlike other organ systems, the hematopoietic system is unique in that active blood production migrates spatially during development, switching dynamically from one site to another over time [16, 25, 118]. The changing sites in development have made it difficult to determine the true origin of the HSC precursor [18]. Some early studies argued that HSCs first arise in the yolk sac from a hematopoietic-endothelial precursor referred to as the hemangioblast [15, 119]. In the last decade, several studies have shown that HSCs originate from the HE cells that line the vasculature, as detailed above. To date, the origin(s) of HSCs in the embryo continue to be debated [18]. The lack of highly specific HSC markers contributes to the difficulty of defining HSC ontogeny [120, 121]. During development, HSCs share many of the same surface markers expressed by endothelial cells [15]. This makes isolation of HSCs from precursor cells challenging and complicates efforts to determine the birthplace of definitive HSCs [122]. Phenotypic profiles that enrich for HSCs also change as HSCs mature within the various sites of hematopoiesis, further complicating their isolation [24, 25, 122] (Table 2). EPCR (CD201) has been postulated to be an HSC-specific marker; however, EPCR is still relatively new and requires further validation [120, 123].

Functional measurement of HSCs during early stages of embryogenesis has also been challenging. Based upon transplantation assays, it has been estimated that, at E10.5, as few as 1 HSC may be present in the embryo and, at E11.5, frequency increases to only 1–2 HSCs per embryo [124, 125]. This highlights a dilemma in our ability to measure HSC activity accurately. Indeed, although the majority of reports show that few nascent HSCs contribute to reconstitution of the adult blood system after transplantation, an elegant study by Ganuza and colleagues has shown with *Confetti*-based labeling of aortic endothelium that hundreds of precursors contribute to life-long hematopoiesis [126]. This finding is consistent with prior observations of large numbers of intra-aortic hematopoietic clusters at E10.5 [42]. In a complementary study, Ganuza et al. also found that repeated exposure of HSCs to transplantation contributed to what authors describe as clonal collapse, indicating that the stress of ex vivo manipulation and transplant drives restriction in clonal diversity [127]. The heterogeneity and limited numbers of HSCs in the embryo can also make identification of epigenetic and transcriptional regulators of HSC function difficult. Recently, single-cell analysis has emerged as a popular method in hematology for characterizing HSC and HSPC populations in the AGM [23, 128, 129]. Collectively, these studies show the potential of new approaches capable of capturing the dynamic nature of EHT and provide possible avenues to overcome the rarity of HSCs in the embryo.

### Analytical Tools to Study Biomechanical Regulators of HSC Function

Despite its importance in HSC specification, we currently lack robust and specific biosensors for quantifying the level of force perceived by HE cells [130]. Biomechanical forces induce a combination of biophysical and biochemical responses [130]. Typically, readouts measuring cellular processes such as calcium uptake, protein production, gene expression, migration, and viability are used as reporters of shear stress [130]. However, a major caveat to this approach is that these readouts are surrogate measures of cellular behaviors that can be regulated independently of force [130]. For example, changes in calcium uptake might not be directly caused by shear stress but rather are induced by unrelated biochemical cues. Additionally, no “standard metric” exists for measuring shear stress [130]. Attempts to develop specific assays have proven difficult, and this remains a significant obstacle in efforts to further understand the impact of shear stress on HSCs [130].

While mouse models allow for rigorous analysis of HSC function, visualization and manipulation of the HSC in its native microenvironment is challenging [131]. Indeed, live-imaging techniques capable of systematically following cellular dynamics long-term in a mouse embryo have yet to be actualized [132]. In addition, our ability to genetically manipulate mouse embryos has greatly improved with the development of tools such as CRISPR/Cas9, but the resources necessary to conduct unbiased genetic screens in mouse embryos significantly limits access to these approaches [131]. On the other hand, zebrafish are able to produce hundreds of transparent embryos amenable to chemical and genetic screens, and high-resolution live imaging has made them an invaluable tool to study embryonic hematopoiesis [133]. Zebrafish have also gained traction as a model for testing hematological disorders. For example, zebrafish engineered to express a fusion protein found in high-risk acute myeloid leukemia (AML) were shown to respond favorably to epigenetic therapies targeting the oncogene and this treatment restored normal hematopoiesis [134, 135]. Functional HSC analysis in zebrafish has advanced in recent years through development of long-term transplantation assays, though historically the zebrafish model has not served as a complete and universal hematology tool [131, 136–138]. As the field seeks more tools to interrogate intracellular and extracellular sources of force in hematopoiesis, invertebrate models are an often overlooked choice. Invertebrate models such as *Drosophila* and *Caenorhabditis elegans* have dominated cell biology for resolution of molecular and cytoskeletal dynamics due to their small size, tissue accessibility, transparency, environmental requirements, and ease of genetic engineering [139]. However, the hematological system in *Drosophila* possesses distinct differences from vertebrates in tissue origination, blood lineages, and stemness/cycling of the HSC equivalent—the prohemocyte. Thus, the fly is likely best reserved for analyses of genetic and molecular regulation of hematopoiesis [131]. Until creative solutions to some of the barriers to detecting and validating HSC emergence in the live embryo are overcome, biomimicry in ex vivo cultures could be the next best approach for interrogating HSC-niche interactions.

While many advancements have been made in tissue engineering, comprehensive characterization of the mechanical landscape surrounding developing HSCs is incomplete [140–142]. This lack of knowledge, along with extensive use of two-dimensional culture, makes recapitulating the native HSC niche problematic. These limitations have been partially addressed by co-culture of HSCs with endothelial cells, bone marrow stromal cells, and/or marrow-inspired extracellular matrix [143, 144]. Indeed, co-culture of differentiating pluripotent stem cells with endothelial cells engineered to express myristoylated Akt and angiocrine factors supportive of hematopoiesis has produced promising evidence that these alternative sources could be used for de novo generation of HSCs ex vivo [14]. As further support for the importance of the signals produced by these niche cells, the same endothelial cells were shown to substantially amplify the HSC population from AGM-derived hematopoietic precursors [143]. Yet, true three-dimensional platforms could better simulate the structural and biochemical properties of the niche [145]. Choi and Harley showed regulation of morphology, proliferation, and fate selection of adult bone marrow-derived HSCs by matrix stiffness and ligand cues within a marrow-inspired extracellular matrix ligand-coated polyacrylamide substrate [144]. Integrin engagement and myosin II activation mediated response to the matrix environment and permitted discrete assessments of the matrix ligands best at promoting HSC maintenance versus differentiation. Similar studies have been conducted with three-dimensional collagen hydrogels to tease apart the effects on HSC self-renewal and differentiation of soluble factors produced by niche cells and even HSCs themselves [146]. More recently, Barnhouse and colleagues have developed endothelial networks in methacrylamide-functionalized gelatin hydrogels to model and interrogate the perivascular secretome [147]. Given the emerging significance of perisinusoidal spaces in the adult bone marrow in HSC maintenance, studies of this kind represent important steps toward identifying both the biomechanical and biochemical cues that regulate HSC cycling. Use of tools such as three-dimensional bioprinting of scaffolds and matrix, along with co-culture of HSCs with niche cells, likely represent the direction that the field must take to adequately test the relevance of biophysical signals in embryonic blood development and apply these lessons toward cell production protocols for clinical use.

## Conclusions

Due to the nascency of mechanobiology to hematology, there remain unexplored aspects of how the extrinsic environment shapes HSC specification and ontogeny, including the key mechanosensors and intracellular mechanotransduction events that translate force into activation of hematopoietic programs of gene expression. Among hemodynamic forces, WSS has been examined most extensively in the last decade. It will be important to further interrogate the roles of hydrostatic pressure and circumferential strain in HSC development as well as develop new ways of measuring the force present at sites of HSC emergence, homing, and expansion. We believe that the use of various model systems that incorporate relevant biomechanical cues, including animal models and in vitro platforms, will elucidate crucial regulators of HSC biology. Future platforms for modeling HSC development and for clinical expansion of HSCs should strive to integrate biomechanical forces and physical features with relevant biochemical factors. A key metric of success for clinical applications would be a design of a system that could expand HSCs while preserving self-renewal potential. Ongoing efforts are also aimed at development of alternative sources of HSCs ex vivo, and such a system would have a distinct function, namely to enable de novo specification of HSCs and/or progenitors capable of producing long-term reconstitution of the blood system. By understanding the signaling activated downstream of force, we can begin to identify those signals that are critical for HSC development and thus inform pharmacological and biomimetic approaches aimed at improving ex vivo HSC specification and expansion.

## Figures and Tables

**Table 1:** Timeline of discovery in the field of hematopoiesis

Year(s)	Scientific breakthrough	References
1956/1957	Bone marrow transplant was first performed by Dr. E. Donnall Thomas	[148]
1961	Bone marrow transplantation generates clonal hematopoietic cells of granulocytic, erythroid, and megakaryocytic origin in the spleen	[149]
1963	Discovery of hematopoietic cells capable of proliferation, self-renewal, and differentiation	[150]
1970	Hematopoietic cells capable of differentiation into granulocytic, erythroid, and megakaryocytic lineages are found to originate in the yolk sac	[151]
1978	Hematopoietic stem cell niche hypothesis was first proposed	[152]
1988, 1991, 1996	Identification of HSC surface markers and isolation of HSCs	[153–155]
1996	Aorta-gonad-mesonephros (AGM) is defined as the site of emergence of HSCs in the embryo	[156]
1996, 1997, 1998	A common precursor of endothelial and hematopoietic stem cells is identified as hemangioblast	[157–159]
1998	HSCs emerge in the AGM as a result of endothelial to hematopoietic transition of endothelium with hematopoietic potential	[26, 37]
1999	HSCs maintain quiescence in the bone marrow during homeostasis	[160]
2000	Fetal liver is determined to be the site of HSC expansion	[161]
2009, 2015	Biomechanical forces associated with blood flow promote HSC emergence in the AGM	[33, 34, 162]
2010	Definitive HSCs emerge from hemogenic endothelium	[27, 28]
2015	Extracellular matrix stiffness regulates HSPC fate in vitro	[163]
2018	Hydrostatic pressure and co-culture with MSC enhances expansion and maintenance of HSPCs	[164]
2020	Cyclic strain in the hemogenic endothelium is necessary for HSPC maintenance, maturation, and proliferation via YAP1 signaling	[103]
2020	Blood flow regulates endothelial cell extrusion in blood vessels	[65]

**Table 2:** Surface markers in HSC development

Embryonic age	Developmental stage	Location	Surface markers
E7.0–8.0	Hemangioblast/hemogenic endothelium	Primitive streak	CD31^+^ [165, 166], CD31^+^CD144^+^ [165, 166], Brachyury^+ −^ [167], CD41^−^ [168–170], CD43^+^ [165], CD45^+^ [36, 171], TER119^+^ [166, 172], Flk1^+^ [18, 55], Scl^−^ [167]
E8.5–10.5	Pre-HSC	Yolk sac, pSp/AGM, placenta	CD41^+^ [15, 170, 173], CD45^+^ [15, 122, 166], CD45^−^ [122], Runx1^+^ [15], CD34^+^ [174], EPCR (CD201^+^) [122, 175], VE-cadherin^+^ (CD144^+^) [15, 122], endomucin^+^ [174], Sca1^−^ [173], Aa4.1^+^ [173]
E11.0–12.5	HSC	Yolk sac, AGM, placenta	Runx1^+^ [15], CD45^+^ [166], VE-cadherin [59], CD41^+ −^ [170], CD34^+ −^ [166], endomucin^+^ [174]
E11.5–16.5	HSC	Placenta, fetal liver	Runx1^+^ [176], VE-cadherin [59], CD45^+^ [177], CD34^+ −^ [174, 178], endomucin^+^ [174], Sca1^+^ [179], Mac1^+^ [179], Aa4.1^+^ [180], CD150^+^CD244^−^CD48^−^ [177, 180]
E17.0–birth	HSC	Bone marrow	Runx1^+^ [176], VE-cadherin^−^ [59], CD45^+^ [177], CD34^−^ [174, 178], endomucin^+^ [174], Sca1^+^ [179], Mac1^−^ [179], Aa4.1^−^ [180], CD150^+^CD244^−^CD48^−^ [177, 180], EPCR (CD201^+^) [181]
